# Prevalence of arterial hypertension in the Krasnoyarsk Krai (Siberia, Russia)

**DOI:** 10.1186/s12872-017-0559-5

**Published:** 2017-05-26

**Authors:** I. P. Artyukhov, Yu. I. Grinshtein, M. M. Petrova, V. V. Shabalin, R. R. Ruf

**Affiliations:** Krasnoyarsk State Medical University named after prof. V. F. Voyno-Yasenetsky, Ministry of Health of the Russian Federation, 660022, St., 1. Partizan Zheleznyak, Krasnoyarsk, Russian Federation

**Keywords:** Arterial hypertension, Prevalence, Epidemiological study, ESSE-RF

## Abstract

**Background:**

To estimate the prevalence, awareness, treatment and control of arterial hypertension among adult inhabitants of Krasnoyarsk Krai using the data from Russian multicenter epidemiological study ESSE-RF (Epidemiological Survey of cardiovascular diSEases in different regions of the Russian Federation).

**Methods:**

The study included 1603 subjects 25 to 64 years old selected by means of systemic multistage stratified randomization among urban and rural inhabitants of Krasnoyarsk Krai recruited between February 2014 and June 2014. Office blood pressure (BP) was measured twice with “Omron” automated BP device on the right arm in the sitting position in presence of medical personnel. Arterial hypertension (HTN) was defined as systolic BP of at least 140 mmHg and/or diastolic BP of at least 90 mmHg or self-reported previous elevated BP registration or use of antihypertensive treatment. Treatment efficacy was defined as percentage of patients achieved the target BP level among those who received antihypertensive medications, and HTN control as percentage of people achieved the target BP level among all hypertensives.

**Results:**

The gender distribution was 652 males (39.4%) and 951 females (60.6%). The average level of systolic BP was 133.4 ± 0.5 mmHg, diastolic BP – 82.9 ± 0.3 mmHg. The average prevalence of HTN was estimated at 49.4% and appeared to be higher than similar parameter based on the data of 10 regions from the ESSE-RF study (44%). The average prevalence of HTN was estimated at 56.3% in males and 43.7% in females. The HTN prevalence in rural community was significantly higher in comparison with urban community (63.4 ± 2.4 vs. 44.2 ± 1.5%, *p* < 0.01). The average rate of HTN awareness in Krasnoyarsk Krai was 77.9% (average Russian value from ESSE-RF study was 73.1%). The average use of antihypertensive treatment, its efficacy and HTN control in Krasnoyarsk Krai were estimated at 59.5%, 31.6% and 18.8%, respectively.

**Conclusions:**

Estimated prevalence of HTN in Krasnoyarsk Krai is higher than the average Russian parameter. The average HTN prevalence among men is higher than in women. The rural inhabitants are more likely to have hypertension compared with urban inhabitants. Despite high levels of HTN awareness and antihypertensive medication intake, the antihypertensive treatment efficacy in Krasnoyarsk Krai appeared to be lower in comparison with average Russian ESSE-RF values.

## Background

Arterial hypertension (HTN) is the most important cardiovascular risk factor. According to World Health Organization experts, high blood pressure (BP) caused about 9.4 million deaths and 162 million years of life lost worldwide in 2010. HTN is also responsible for 50% of cases of coronary heart disease, stroke and heart failure, 18% of all deaths and 40% of deaths among patients with diabetes mellitus [1]. Costs for HTN and HTN-associated diseases treatment can reach 25% of all costs of healthcare systems in Eastern European countries [1].

Approximately 40% of adults aged 25 and over have HTN. By the age of 80 the rate of people suffering from HTN can reach 90% [1].

While prevalence of HTN remains relatively stable in highly developed countries (i.e. in the USA it stayed approximately 29% for 9 years from 2003 to 2012 [2]), in developing countries, low and middle income countries (such as India, Pakistan, Brazil, Argentina or China) increasing amount of people with hypertension is becoming a significant social and economic issue [3].

During the prospective study in Iran (median follow-up of 9.6 years), they revealed increasing incidence of isolated systolic and isolated diastolic hypertension (about 0.5% and 1% per year, respectively) [4].

In South Korea, HTN prevalence elevation was also registered from 1998 to 2012 [5].

Few data exist on gender influence on HTN prevalence. Particularly, higher HTN prevalence in males compared to females has been revealed in Nepal [6]. Researchers from Iran showed that females have significantly lower risk of developing hypertension [4]. The study from Barbados suggests however that smoking, insufficient physical activity, obesity and excessive alcohol intake often depend on gender, but there is no significant dependence between gender and high BP [7].

Some studies [8, 9] have demonstrated differences between urban and rural population in prevalence, awareness, treatment efficacy and control of HTN. In rural population prevalence of HTN often appears higher, while awareness, treatment efficacy and control of HTN are usually lower than in urban population.

The purpose of the study was to estimate HTN prevalence and awareness, parameters of treatment with antihypertensives, and BP control in population of Krasnoyarsk city and Beryozovsky District of Krasnoyarsk Krai within the scope of the Russian multicenter study ESSE-RF.

## Methods

A total of 1603 people aged 25–64 from the population served by four outpatient clinics of Krasnoyarsk and Beryozovka territorial hospital were enrolled into the study by means of systematic stratified multistage randomization [10]. After informed consent signing all participants of the study filled in a detailed questionnaire and underwent office BP measurement. The questionnaire consisted of 12 modules based on adapted international methods and included questions on presence of hypertension, disease awareness and antihypertensive medication*.*

BP level was measured twice within 2–3 min after a 5-min rest with Omron automated BP monitor on the patient’s right arm in the presence of medical personnel. HTN was diagnosed if measured systolic BP (SBP) was higher than 140 mmHg and/or diastolic BP (DBP) higher than 90 mmHg, and if the questionnaire revealed that the patient had ever had high BP and/or took antihypertensive medications.

Treatment efficacy was calculated as a percentage of patients that reached target BP levels among patients who took antihypertensive medications. BP control was calculated as a percentage of patients with target BP levels among all patients with hypertension.

Statistical calculations were performed with IBM SPSS v 22 and LibreOffice Calc v 5. With Lilliefors test, we found numeric parameters distributions not normal (null-hypothesis rejected; *p* < 0.001) and described them as median (25%-quartile; 75%-quartile). Nominal parameters described as percentage (%) ± 95% confidence interval (CI). Group disparities were checked for significance with Mann-Whithey and Kruskal-Wallis test for numeric parameters and Chi-square test for nominal parameters. We considered differences significant at *p* ≤ 0.01.

## Results

Our sample of Krasnoyarsk Krai population included 652 males (39.4%) and 951 females (60.6%) with SBP distributed as 131.0 (119.0; 144.5) mmHg and DBP as 82.0 (74.5; 90.5) mmHg. These figures are slightly higher in comparison with averages all over Russia according to the ESSE-RF study (average 130.7 ± 0.10 and 81.6 ± 0.50 mmHg respectively) [11]. SBP and DBP in males (137.0 (125.0; 148.0) and 84.5 (76.5; 91.5) mmHg respectively) appeared significantly higher (H_0_ rejected, *p* = 0.01) than in females (126.5 (115.0; 141.5) and 81.0 (72.5; 89.5) mmHg, respectively); for details and age dependencies see Table 1.

**Table 1:** BP levels, HTN prevalence and HTN awareness depending on age and gender

Age group (years)	Male		Female		Total	
Levels of Systolic Blood Pressure (SBP) in different age groups (mmHg, median (Q25; Q75)						
	SBP	Q25; Q75	SBP	Q25; Q75	SBP	Q25; Q75
25–34	127.5	119.5; 137.5	116.0	109.0; 125.0	120.5	111.5; 130.5
35–44	134.3	124.3; 142.8	121.5	113.0; 132.0	126.0	116.0; 138.5
45–54	140.0	129.5; 148.5	130.5	118.5; 142.5	134.0	122.0; 145.0
55–64	144.0	135.0; 158.5	141.0	130.0; 156.5	142.5	132.5; 157.0
Levels of Diastolic Blood Pressure (DBP) in different age groups (mmHg, median (Q25; Q75)						
	DBP	Q25; Q75	DBP	Q25; Q75	DBP	Q25; Q75
25–34	78.5	73.0; 84.5	74.5	69.0; 81.0	76.0	71.0; 82.5
35–44	83.5	76.3; 91.0	79.5	72.5; 88.0	82.0	74.0; 89.0
45–54	88.5	81.5; 93.0	82.0	72.5; 90.0	85.0	76.0; 91.5
55–64	88.0	80.5; 95.5	86.0	80.0; 94.0	87.0	80.0; 95.0
Hypertension prevalence in different age groups (% ± 95% CI)						
	% (n)	95% CI	% (n)	95% CI	% (n)	95% CI
25–34	27.0 (*n* = 44)	6.86	10.8 (*n* = 25)	3.92	17.5 (*n* = 69)	3.72
35–44	43.3 (*n* = 52)	8.82	32.1 (*n* = 62)	6.66	36.4 (*n* = 114)	5.29
45–54	71.0 (*n* = 103)	7.39	53.0 (*n* = 134)	6.15	59.5 (*n* = 237)	4.90
55–64	79.9 (*n* = 143)	5.88	76.7 (*n* = 198)	5.15	78.0 (*n* = 341)	3.92
Hypertension awareness in different age groups (% ± 95% CI)						
	% (n)	95% CI	% (n)	95% CI	% (n)	95% CI
25–34	70.5 (*n* = 31)	6.27	76.0 (*n* = 19)	4.51	72.5 (*n* = 50)	10.58
35–44	82.7 (*n* = 43)	7.25	79.0 (*n* = 49)	5.49	80.7 (*n* = 92)	7.25
45–54	73.8 (*n* = 76)	6.86	79.9 (*n* = 107)	4.70	77.2 (*n* = 183)	5.29
55–64	80.4 (*n* = 115)	5.88	77.3 (*n* = 153)	5.10	78.6 (*n* = 268)	4.31

A significant (H_0_ rejected, *p* = 0.01) trend of increasing SBP with increasing age was revealed both in males and females, while DBP slowed its growth with increasing age, and did not grow at all in males aged 55–64 years.

Average HTN prevalence in Krasnoyarsk Krai appeared rather high (49.4%). It is higher than the average for 10 regions of Russia included in the ESSE-RF study (44.0%). In addition, we found significantly (χ^2^ for trend; *p* < 0.001) higher HTN prevalence in males (56.3%) than in females (43.7%). This difference is especially significant (more than 2 times) in the youngest age group (25–34 years old) and flattens in older age (79.9% in males and 76.7% in females in the group of patients aged 55–64) because of increasing number of hypertensive patients among females (see Table 1).

HTN prevalence also significantly (χ^2^ for trend; *p* < 0.001) depends on the place of living both in the general (63.4 ± 4.70% in rural vs 44.2 ± 2.94% in urban) and in the gender groups (65.0 ± 6.66% vs 52.2 ± 4.90% in males and 62.0 ± 6.47% vs 39.6 ± 3.53% in females, respectively).

Average HTN awareness in Krasnoyarsk Krai is 77.9%. We found it rather high in all age groups. However, among patients younger than the age of 55, females are better aware of their disease than males (see Table 1).

To estimate antihypertensive therapy effectiveness, we calculated self-reported use of antihypertensive medications and percentage of patients who achieved target BP both among patients taking antihypertensive medications and among all hypertensive patients. In Krasnoyarsk Krai, 59.5% of patients aged 25–64 take antihypertensive medications. Antihypertensive therapy appeared efficient (target BP levels achieved) in 31.6% of treated hypertensives and in 18.8% of all hypertensive patients. For more details on incidence of antihypertensive medications intake, reaching target BP levels and their dependence on gender and age see Table 2.

**Table 2:** The use of antihypertensive medications and the rate of hypertension control depending on age and gender

Age group in years	Male		Female		Total	
The use of antihypertensive medications (% ± 95% CI)						
	% (n)	95% CI	% (n)	95% CI	% (n)	95% CI
25–34	22.7% (*n* = 10)	12.35	44.0% (*n* = 11)	19.40	30.4% (*n* = 21)	10.78
35–44	28.8% (*n* = 15)	12.35	50.0% (*n* = 31)	12.35	40.4% (*n* = 46)	9.02
45–54	49.5% (*n* = 51)	9.60	66.4% (*n* = 89)	8.04	59.1% (*n* = 140)	6.27
55–64	65.0% (*n* = 93)	7.84	77.3% (*n* = 153)	5.88	72.1% (*n* = 246)	4.70
Percentage of patients achieved target BP among hypertensives receiving antihypertensive medications (% ± 95% CI)						
	% (n)	95% CI	% (n)	95% CI	% (n)	95% CI
25–34	70.0% (*n* = 7)	28.42	45.5% (*n* = 5)	29.40	57.1% (*n* = 12)	21.17
35–44	40.0% (*n* = 6)	24.89	41.9% (*n* = 13)	17.44	41.3% (*n* = 19)	14.31
45–54	27.5% (*n* = 14)	12.35	47.2% (*n* = 42)	10.39	40.0% (*n* = 56)	8.04
55–64	20.4% (*n* = 19)	8.23	24.2% (*n* = 37)	6.86	22.8% (*n* = 56)	5.29
The rate of hypertension control among all hypertensives (% ± 95% CI)						
	% (n)	95% CI	% (n)	95% CI	% (n)	95% CI
25–34	15.9% (*n* = 7)	10.80	20.0% (*n* = 5)	15.68	17.4% (*n* = 12)	9.02
35–44	11.5% (*n* = 6)	8.68	21.0% (*n* = 13)	10.19	16.7% (*n* = 19)	6.86
45–54	13.6% (*n* = 14)	6.62	31.3% (*n* = 42)	7.84	23.6% (*n* = 56)	5.49
55–64	13.3% (*n* = 19)	5.57	18.7% (*n* = 37)	5.49	16.4% (*n* = 56)	3.92

Percentage of patients taking antihypertensive medications noticeably grows with age (from 30.4% to 72.1% of hypertensive patients), more in females than in males (χ^2^ for trend; *p* < 0.001). Target BP levels achievement demonstrates another dependence: the older the patients are, the smaller percentage of patients reaches target levels of BP (from 57.1% to 22.8%; χ^2^ for trend; *p* < 0.001). Again, females reach target BP more often than males, except for the 25–34 years group.

In comparison with all-Russian data from 10 regions included in the ESSE-RF study in Krasnoyarsk Krai, more patients take antihypertensive medications (49.4% vs 39.5% of males; 67.8% vs 60.9% of females, and 59% vs 53.1% in general), but the efficacy of antihypertensive treatment in our region is lower than in the rest of Russia (27.2% vs 41.4% males, 34.2% vs 53.5% females, and 31.6% vs 49.1% in general).

The educational background of patients not only has significant influence upon their BP level (H_0_ rejected, *p* = 0.01), but also antihypertensive treatment efficacy (χ^2^ for trend; *p* = 0.007) and the whole population’s prevalence of HTN (χ^2^ for trend; *p* < 0.001). However, incidence of antihypertensive medications intake and HTN awareness do not depend significantly on the educational background (for details, see Tables 3, 4).

**Table 3:** Levels of BP, hypertension prevalence and hypertension awareness depending on education level

Education level	Male		Female		Total	
Levels of Systolic Blood Pressure (SBP) depending on education level (mmHg, median (Q25; Q75)						
	SBP	Q25; Q75	SBP	Q25; Q75	SBP	Q25; Q75
Higher education	133.0	122.5; 142.5	121.5	112.0; 135.5	125.5	115.5; 139.0
Secondary education	139.0	127.3; 150.0	133.0	120.8; 146.0	136.0	122.5; 148.0
Primary education	141.5	126.0; 155.5	133.3	121.0; 142.5	140.0	125.5; 149.5
Levels of Diastolic Blood Pressure (DBP) depending on education level (mmHg, median (Q25; Q75)						
	DBP	Q25; Q75	DBP	Q25; Q75	DBP	Q25; Q75
Higher education	82.5	76.0; 90.0	77.5	70.5; 86.5	80.0	72.0; 87.5
Secondary education	85.5	77.5; 92.5	83.0	76.0; 91.5	84.5	77.0; 92.0
Primary education	86.5	76.5; 94.5	83.5	77.0; 90.0	85.5	76.5; 91.0
Hypertension prevalence depending on education level (% ± 95% CI)						
	% (n)	95% CI	% (n)	95% CI	% (n)	95% CI
Higher education	45.6 (*n* = 104)	6.47	32.4 (*n* = 156)	4.12	36.7 (*n* = 260)	3.53
Secondary education	62.7 (*n* = 208)	5.10	57.5 (*n* = 246)	4.70	59.7 (*n* = 454)	3.53
Primary education	63.8 (*n* = 30)	13.70	65.4 (*n* = 17)	18.23	64.4 (*n* = 47)	10.98
Hypertension awareness depending on education level (% ± 95% CI)						
	% (n)	95% CI	% (n)	95% CI	% (n)	95% CI
Higher education	80.8 (*n* = 84)	5.10	83.4 (*n* = 120)	3.33	82.7 (*n* = 204)	2.74
Secondary education	75.5 (*n* = 157)	4.51	81.5 (*n* = 197)	3.72	79.5 (*n* = 354)	2.94
Primary education	80.0 (*n* = 24)	10.19	61.5 (*n* = 11)	18.62	76.7 (*n* = 35)	9.60

**Table 4:** The use of antihypertensive medications and the rate of hypertension control depending on education level

Education level	Male		Female		Total	
The use of antihypertensive medications depending on education level (% ± 95% CI)						
	% (n)	95% CI	% (n)	95% CI	% (n)	95% CI
Higher education	53.8 (*n* = 56)	9.60	62.2 (*n* = 97)	7.64	58.8 (*n* = 153)	6.08
Secondary education	48.1 (*n* = 100)	6.86	71.5 (*n* = 176)	5.68	60.8 (*n* = 276)	4.51
Primary education	43.3 (*n* = 13)	17.84	64.7 (*n* = 11)	22.74	51.1 (*n* = 24)	14.31
Percentage of hypertensives achieved target BP among receiving antihypertensive treatment depending on education level (% ± 95% CI)						
	% (n)	95% CI	% (n)	95% CI	% (n)	95% CI
Higher education	35.7 (*n* = 20)	12.54	44.3 (*n* = 43)	9.80	41.2 (*n* = 63)	7.84
Secondary education	23.0 (*n* = 23)	8.23	28.4 (*n* = 50)	6.66	26.4 (*n* = 73)	5.29
Primary education	23.1 (*n* = 3)	22.93	36.4 (*n* = 4)	28.42	29.2 (*n* = 7)	18.23
The rate of hypertension control among all hypertensives depending on education level (% ± 95% CI)						
	% (n)	95% CI	% (n)	95% CI	% (n)	95% CI
Higher education	19.2% (*n* = 20)	7.64	27.6% (*n* = 43)	7.06	24.2% (*n* = 63)	5.10
Secondary education	11.1% (*n* = 23)	4.31	20.3% (*n* = 50)	5.10	16.1% (*n* = 73)	3.33
Primary education	10.0% (*n* = 3)	10.78	23.5% (*n* = 4)	20.19	14.9% (*n* = 7)	10.19

## Discussion

According to this study’s results, the levels of mean systolic and diastolic BP among representative sample of adults aged 25 to 64 years old in Krasnoyarsk Krai appeared somewhat higher compared with corresponding Russian national average figures taken from the ESSE-RF study.

Average HTN prevalence in Krasnoyarsk Krai (49.4%) appeared to be significantly higher than average for 10 regions of Russia included in the ESSE-RF study (44.0%). Most studies in Western countries report considerably lower prevalence of HTN: 19.5% in Canada, 29% in USA, 30% in England [12], 42.6% in Spain [13], as well as in China (32.5%) [14]. Nevertheless, certain districts of Serbia showed higher HTN prevalence (53%) [15].

Noteworthy, significantly higher prevalence of HTN was found in males than in females (56.3% vs 43.7%). It is associated with modern national and international tendency. However, this difference flattens in the older age (55–64 years old). Results of this study have clearly demonstrated dependence of HTN prevalence on the place of living: rural population had significantly higher prevalence of HTN in general and in both gender groups.

Average HTN awareness in Krasnoyarsk Krai was 77.9%. It was found rather high in all age groups. Exceeding the average national level of awareness (73.1%), this parameter corresponds well with the data from developed Western countries (81% in USA, 83% in Canada, 65% in England) [12]. HTN awareness in Krasnoyarsk Krai appeared to be much higher than in low-income and middle-income countries (40.8–50.2%, according to PURE Study; from 33.5% to 69.0%, according to NHLBI/UHG Network) [16, 17]. Unfortunately, the efficacy of antihypertensive treatment leaves much to be desired. While in Krasnoyarsk Krai 59.5% patients aged 25–64 receive antihypertensive medications, only 31.6% of treated hypertensives (and 18.8% of all hypertensive population) achieve target BP levels. It seems to be that reasons for inadequate level of BP control include insufficient patients’ compliance (inaccuracy in following prescribed mode of medication intake), non-optimal choice of antihypertensive therapy, its combination and dosing by physicians. Noteworthy, the effectiveness of HTN treatment in highly industrialized countries is continuously growing (the rate of hypertension control is reported to be 66% in Canada [12] and up to 70% in selected population in the USA [2]).

## Conclusions

In view of this, the results of this epidemiological study indicate that HTN prevalence among adults in Krasnoyarsk Krai is rather high and exceeds the corresponding average Russian HTN rate. Prevalence of HTN is significantly higher in males than in females excluding the age group 55–64 years, in which this difference is disappearing. Rural inhabitants are more likely to have HTN than urban citizens (both in males and females, as well as in the whole population). Despite rather high levels of HTN awareness and antihypertensive treatment, treatment efficacy in target BP achievement appeared to be lower in Krasnoyarsk Krai as compared to average Russian ESSE-RF data.

### Limitations

Being a part of ESSE-RF study, our study was limited to research 25–64 years old age group as the most employable part of population. Diagnosis of hypertension was based on measures and history taken at a single visit, but this approach is similar to that of many epidemiological studies.

## Abbreviations

BP: Blood pressure
DPM: diastolic blood pressure
ESSE-RF: Epidemiological Survey of cardiovascular diSEases in different regions of the Russian Federation
HTN: Hypertension
SBP: systolic blood pressure.
